# Efficacy and Function of Feathers, Hair, and Glabrous Skin in the Thermoregulation Strategies of Domestic Animals

**DOI:** 10.3390/ani11123472

**Published:** 2021-12-06

**Authors:** Daniel Mota-Rojas, Cristiane Gonçalves Titto, Ana de Mira Geraldo, Julio Martínez-Burnes, Jocelyn Gómez, Ismael Hernández-Ávalos, Alejandro Casas, Adriana Domínguez, Nancy José, Aldo Bertoni, Brenda Reyes, Alfredo M. F. Pereira

**Affiliations:** 1 Neurophysiology, Behavior and Animal Welfare Assessment, DPAA, Universidad Autónoma Metropolitana (UAM), Unidad Xochimilco, Mexico City 04960, Mexico; jocelyn.gomez.ilp@gmail.com (J.G.); ale0164g@hotmail.com (A.C.); mvz.freena@gmail.com (A.D.); nancyjosenutricion2017@gmail.com (N.J.); aldo_bm@hotmail.com (A.B.); breyess_20@yahoo.com.mx (B.R.); 2 Laboratório de Biometeorologia e Etologia, FZEA-USP, Faculdade de Zootecnia e Engenharia de Alimentos, Universidade de São Paulo, Pirassununga 13635-900, SP, Brazil; crisgtitto@usp.br; 3 Mediterranean Institute for Agriculture, Environment and Development (MED), Institute for Advanced Studies and Research, Universidade de Évora, Pólo da Mitra, Ap. 94, 7006-554 Évora, Portugal; ageraldo@uevora.pt; 4 Animal Health Group, Facultad de Medicina Veterinaria y Zootecnia, Universidad Autónoma de Tamaulipas, Victoria City 87000, Mexico; jmburnes@docentes.uat.edu.mx; 5 Facultad de Estudios Superiores Cuautitlán, Universidad Nacional Autónoma de México (UNAM), Cuautitlan Izcalli 54714, Mexico; mvziha@hotmail.com

**Keywords:** birds, behavior, thermoregulation, skin, glabrous skin, hair, feathers

## Abstract

**Simple Summary:**

Animals adopt several strategies to regulate their body temperature by promoting heat loss or gain in hot and cold environments, respectively. This mechanism of heat loss or production is performed in thermal windows. A thermal window is a structure where many blood capillaries facilitate thermal exchange in this region. The presence of feathers, hair, or glabrous (hairless) skin and their structural characteristics greatly influence each species’ capacity to maintain thermal comfort. This factor needs to be considered when implementing new monitoring or measuring techniques such as infrared thermography since interpretations may vary due to the presence or absence of these structures. It is essential to recognize the effects of glabrous skin, hair, and feathers on thermoregulation to identify species-specific thermal windows that allow accurate evaluations of the thermal state of domestic animals.

**Abstract:**

The objective of this review is to describe and analyze the effect of feathers, hair, and glabrous (hairless) skin on the thermoregulation of domestic and endotherm animals, especially concerning the uses and scope of infrared thermography (IRT), scientific findings on heat and cold stress, and differences among species of domestic animals. Clinical medicine considers thermoregulation a mechanism that allows animals to adapt to varying thermal environmental conditions, a process in which the presence of feathers, hair, or glabrous skin influences heat loss or heat retention, respectively, under hot and cold environmental conditions. Evaluating body temperature provides vital information on an individual’s physiological state and health status since variations in euthermia maintenance in vertebrates reflect a significant cellular metabolism deviation that needs to be assessed and quantified. IRT is a non-invasive tool for evaluating thermal responses under thermal stress conditions in animals, where the presence or absence of feathers, hair, and glabrous skin can affect readings and the differences detected. Therefore, anatomical regions, the characteristics of feathers, hair, glabrous skin such as structure, length, color, and extension, and strategies for dissipating or retaining heat together constitute a broad area of opportunity for future research into the phenomena of dermal thermoregulation in domestic species.

## 1. Introduction

Thermoregulation is a process that responds to specific environmental conditions, such as heat or cold and can entail significant energy expenditures by individuals to maintain temperature homeostasis [1,2,3]. These processes include a series of circulatory, physiological, behavioral, and metabolic adjustments to achieve the loss or retention of heat necessary to maintain correct cellular homeostasis and reduce the consequences that thermal stress implies [4]. Thermal stress is an external condition that produces a strain in the biological functions of animals and is triggered when the environmental conditions exceed the critical range of temperature, requiring an increase in their basal metabolic rate to thermoregulate [5].

This critical temperature range is known as the thermoneutrality zone, which is a “range of environmental temperature, specie-specific, at which thermoregulation is achieved solely by control of sensible heat without regulatory changes in metabolic heat production or evaporative heat loss” [6].

Thermoneutrality is often associated with the thermal comfort zone; however, comfort is defined “in terms of perception and the state of mind that expresses satisfaction with the thermal conditions of the environment” [7].

The responses triggered by animals outside the mentioned thermal zones involve various pathways in which the central nervous system (CNS) participates through the hypothalamus and the brainstem. On the other hand, the peripheral response is initiated by activating receptors specialized in recognizing thermal stimuli and the secretion of neurotransmitters secreted by the hypothalamus [8]. Among these thermoreceptors, the Transient Receptor Potential (TRP) ion channels are the main receptors implicated in perceiving specific ranges of temperatures [9]. Within the TRP family, two groups of ion channels are recognized for the perception of warm or hot temperatures (e.g., TRPV1-4, TRPM2, TRPM3, TRPM5) and cool or cold stimuli (e.g., TRPM8, TRPA1, and TRPC5) [10]. The depolarization and action potentials produced by these receptors reach superior thermoregulatory centers in the hypothalamus, leading to physiological, behavioral, internal, and external mechanisms necessary to reach homeothermy [11,12].

**Figure 1:**
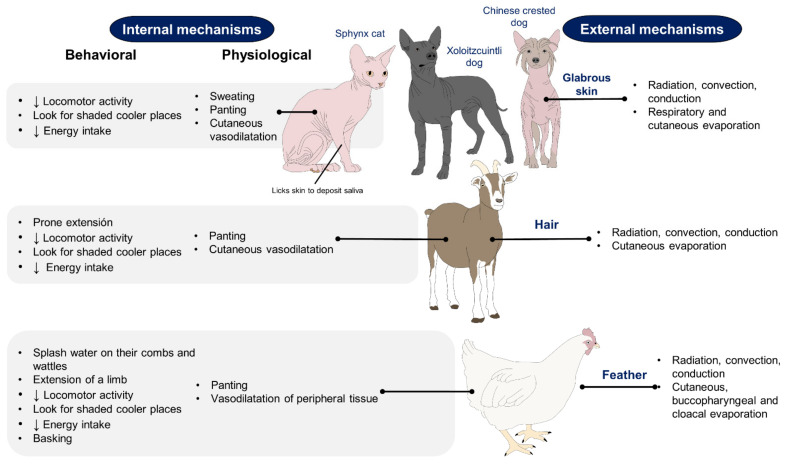
Compensatory mechanisms that animals with glabrous skin, hair, or feathers develop for hyperthermia avoidance. The morphological differences between species influence their behaviors and physiological responses to dissipate heat. Sphynx cats, Xoloitzcuintli, and Chinese crested dogs (seen in figure) sweat in the same way as other domestic animals. However, due to the bare skin, insensible perspiration is higher than other animals with hair or full coat and are predisposed to acute peripheral vasomotor responses to thermoregulate [15,16,17].

To avoid thermal stress, animals use three main mechanisms to thermoregulate [11,13]. In the first instance, heat stress occurs in extreme environmental conditions, such as hot environments, resulting in an imbalance between internal demands and the environment, where the capacity to dissipate heat is altered [14]. During heat stress, physiological mechanisms promote heat loss through cutaneous vasodilation and sensible heat loss by conduction, convection, and radiation due to the thermal gradient between animal and environment. Sweating and panting also increase evaporative heat loss when exposed to hot temperatures [15,16,17] (Figure 1). These mechanisms are essential, particularly for the Sphynx cat, the Xoloitzcuintli dog (Mexican hairless dog), and the Chinese Crested dog, which breeds characterized by a partial or entire absence of hair, classified as animals with glabrous skin [18]. In the Sphynx cat, the presence of small and curved hair follicles with infundibulum and a hair bulb is recognized, but these follicles are in the continuous anagen phase, producing dysplastic hairs that cannot penetrate the epidermis and are not able to form a proper coat [19]. On the other hand, in the Chinese crested dog, an autosomal dominant trait generates ectodermal dysplasia, deformities in the hair follicles, and condensation of mesenchymal cells, restricting the hair growth to the head, extremities, and tail [19,20].

**Figure 2:**
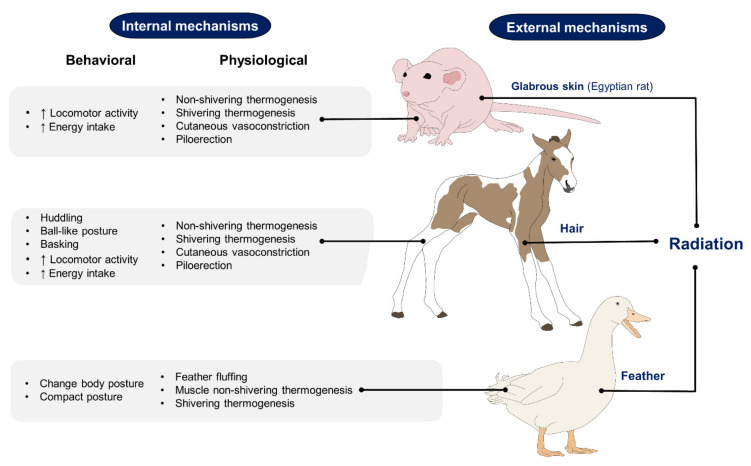
Compensatory mechanisms that animals with glabrous skin, hair, or feathers develop for hypothermia avoidance. The morphological differences between species influence their behaviors and physiological responses to minimize heat loss [26,27,28].

Although these areas of glabrous skin have thicker epidermis and a more compact stratum corneum [18], the absence of hair can alter the capacity of an animal to thermoregulate. Since hair is a physical barrier that intervenes in heat loss or heat retention, hairless animals are more sensitive to extreme temperatures and are predisposed to consequences such as sunburn when exposed to direct sun radiation [21] and acute vasomotor responses [22]. Additionally, in these species, particularly in the Xoloitzcuintli and the Crested dog, structural and functional defects are also present in the sweat glands [20], motivating them to resort to specific behaviors such as saliva spreading onto the body surface to dissipate heat through evaporation [23]. In contrast, responses to avoid hypothermia are activated when animals are under cold stress, where the temperatures are below the comfort zone, and the organism requires to increase its energy intake or metabolic production to maintain the body temperature [24]. The main mechanisms to preserve or produce heat include increased activity, postural changes, shivering, and in some cases, non-shivering thermogenesis, peripheral vasoconstriction, piloerection, among others (Figure 2) [13,25,26,27,28].

Some studies have suggested that infrared thermography (IRT) can aid in identifying responses to heat or cold by evaluating surface temperatures [29,30,31]. This technique detects changes in the blood flow of the microvasculature in response to pathophysiological or environmental events such as heat or cold stress. As explained earlier, both situations trigger vasomotor responses of vasodilation or vasoconstriction, with the subsequent increase or decrease in the amount of heat dissipated and detected by thermography [32,33]. However, the research conducted to date presents contrasting results because the regions useful for assessing temperature differ among animal species [28,29,30]. Only limited information is available on thermal differences related to different types of body coverings—feathers, hair, glabrous skin—and extreme temperatures; for example, the relation between the consumption of energy resources and the type of brown adipose tissue (BAT) when animals are exposed to cold [34], or circulatory changes in response to environmental heat [35].

This review aims to describe and analyze thermoregulation mechanisms in domestic animals related to the effect of feathers, hair, and glabrous skin and how these coverings can influence IRT studies. In addition, we discuss the uses, scope, and scientific findings related to the IRT technique under conditions of stress caused by cold or heat.

## 2. Differences in Thermoregulation Strategies between Animals with Feathers, Hair, and Glabrous Skin

Changes in environmental temperature directly affect animals’ activity levels and behavior [36], two of the main factors that determine their dietary caloric needs and the amounts of resources they require. In some species, these energy resources (reserves) accumulate in fat, but in smaller species, with reduced body surfaces, this strategy is less efficient, so they depend on their metabolic rate to thermoregulate their bodies [37]. In the following paragraphs, we analyze thermoregulation strategies and how they differ among species.

### 2.1. Birds

The specific strategies that animals with feathers (birds) adopt to maintain their body temperature within a normal range contrast to those types of mammals [38] because they depend on plumage or enhanced respiration that increases their metabolic rate. In addition to their essential role in flight, feathers serve as insulation, which helps explain why birds have the highest body temperatures of all vertebrate species. This mechanism allows them to achieve thermoneutrality in cold weather, but in hot weather, it increases their propensity to suffer heat stress, a condition that requires additional mechanisms to maintain homeostasis. Two other factors that influence the regulatory capacity of birds are species and variations in their plumage in different body regions [39]. Respiratory rate in birds called gular fluttering is performed according to the resonance frequency of the bird so as not to expend so much energy. It wastes energy, but the net result is favorable to heat loss. In general, birds conserve heat by trapping air between their skin and feathers to create a barrier that prevents heat loss [40] by reducing thermal conductance; that is, the amount of heat that an animal dissipates into the environment [41]. To the authors’ knowledge, seasonal changes in plumage insulation have not been measured. However, some studies revealed that the feather cover in several species of birds was heavier during the winter than in the summer.

**Figure 3:**
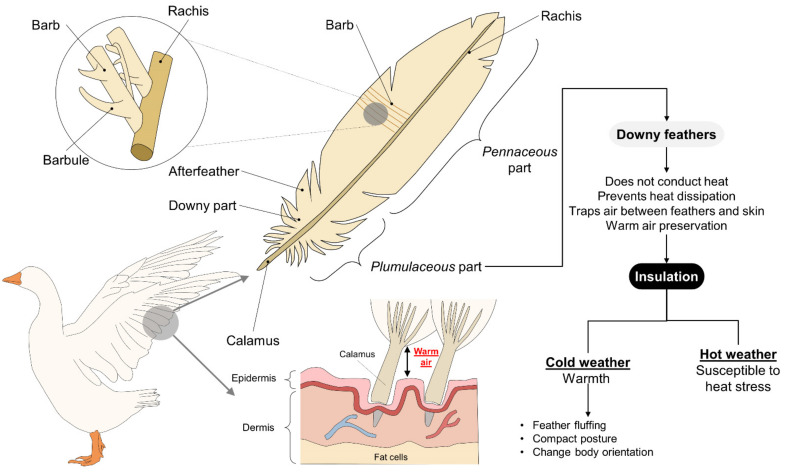
The structure of feathers and thermoregulation in birds: The plumulaceous part and downy feathers are the main structures involved in thermobalance. When exposed to cold weather, birds adopt behaviors such as fluffing their feathers to trap air between them and their skin. This prevents heat dissipation and preserves warm air that raises their body temperature. However, during hot weather, the feathers’ insulating function leaves birds susceptible to heat stress by reducing their ability to thermoregulate efficiently.

Nonetheless, Metabolic studies have not suggested substantial seasonal insulative changes in birds. Irving [42] pointed out that feathers are adapted primarily for flight, limiting insulative modification. The structure of a bird’s feathers includes contour feathers (*penna contorna*) composed of a proximal downy (plumulaceous) part in adults, and a distal, or pennaceous, part. The downy section is the central part involved in thermal insulation [43], as shown in Figure 3. Studies have demonstrated that the microstructure of feathers and their adaptive-evolutionary changes depend on the species, habitat, and size of birds, with smaller species having longer feathers that provide greater thermal insulation capacity than larger ones [44]. One example of the morphological differences among species and habitats comes from a comparison of terrestrial and aquatic birds, for the size, length of barbule, and density of the nodus of the feathers of terrestrial species are all greater than in aquatic birds. At the same time, the latter require a larger plumulaceous layer. These differences reveal how the morphology of the downy part and afterfeathers had to adapt to the demands of distinct environmental conditions [45]. Those authors observed a similar phenomenon in passerines at low temperatures, as the feathers of those birds have a longer proximal (plumulaceous) part with low barb and pennaceous barbule densities [46]. There are also reports that the plumulaceous section of aquatic birds’ feathers is shorter, perhaps due to the need to limit the amount of air that their feathers retain, and so control the buoyancy of their bodies.

Concerning environmental conditions, the first post-hatch week in broilers is a critical time for their thermoregulation. Temperatures between 32 and 35 °C induce hyperthermia with consequent loss of weight gain; in contrast, when exposed to cold temperatures (20 to 25 °C), hypothermia can lead to a predisposition to respiratory diseases and mortality. In 80 1-day-old male chickens of the Cobb strain, an environmental temperature of 20 °C reduced the cloacal temperature (*p* < 0.01), and in these animals, vasomotor responses were activated mainly in featherless skin, where venous anastomoses contribute to 17 an 83% of blood flow that is thermoregulator [47]. These regions (the face and legs) are considered heat radiation zone that require hypothalamic integration, sympathetic activity, and vasomotor response to produce effects such as peripheral vasoconstriction to reduce their temperature by 67 to 71% in temperatures of 10 to 15 °C [48].

These featherless areas are a critical region for thermoregulation of post-hatched birds; however, this is not the only difference between young birds and adults. The origin of the feathers begins from the embryonic tissue during their first days of life. Unlike adult animals, in embryonic chicken down feathers, the branches only include the ramus and the barbule regions, whereas, in adults, the rachis, the ramus, and the barbules are present [49]. After birth, the downy feathers transform into juvenile plumulaceus feathers during the first week of life [50]. A recent study by Barve et al. [44] examined 1715 Himalayan passerines and demonstrated that a high proportion of down in adults is an evolutionary characteristic in cold environments that provides better heat conservation. Tropical species, in contrast, cannot increase their metabolism to produce heat, so they adapt to cold temperatures through a broader threshold of thermoneutrality and a much lower rate of heat loss or conductance than other species [41].

These observations show the role that feathers have during heat or cold stress. In the first case, the insulator property of feathers hinders heat loss during hot temperatures, while in the latter, feathers contribute to heat insulation [51]. Peguri and Coon [52] investigated the presence or absence of feathers in 59-week-old chickens from the white leghorn strain. In these animals, the feather’s effect on the performance and food intake was determined in hot and cold climates (12.8 °C and 33.9 °C, respectively), in animals where feathers were removed in 0, 50, or 100%. Hens without feathers exposed to low temperatures obtained low egg weights and increases in feed consumption (*p* < 0.05). This study demonstrated the importance of thermoneutrality in domestic animals and the productive consequences of not being kept in suitable temperatures and conditions [53].

Feather color also influences birds’ thermoregulating capacity by producing differences at the morphological level. Koskenpato et al. [54] compared the density and structure of the feathers of pale gray owls (*Strix aluco*) and brown, tawny owls. In the former, they found that the plumulaceous portion of the back of the feathers was longer and denser than in the brown-colored owls. The above denotes greater morph-specific adaptability to cold climates due to the respective properties of dark surfaces (melanistic forms) and light ones, where the former absorb more solar radiation than the last, reflect less radiation, and the heat load is more significant [55].

Another characteristic to be considered in avian species is the absence of brown adipose tissue (BAT). A critical mechanism of thermogenesis in cold climates is shivering [38], an action generated by the prominent skeletal musculature of the pectoral region. The pectoralis and supracoracoideus muscles, for example, are considered thermogenic organs because they facilitate the utilization of oxygen and energy substrates in addition to their crucial role in enabling and facilitating flight [56].

During exposure to cold climates, one of the first strategies that birds employ consists of fluffing their feathers because the greater thickness of the plumage increases the insulation, allowing them to maintain thermal balance [57]. Studies of greater snow goose goslings have demonstrated that adjustments such as changes in body orientation concerning wind can reduce thermoregulatory costs by modifying birds’ thermal environment [58]. Larger birds in artic appear to have a critical temperature so low that they can live in arctic winter without special metabolic effort, like the case of *Lagopus lagopus* or *Larus hyperboreus*. Likewise, birds can compact their posture to reduce their surface area and, therefore, heat loss. For example, in domestic fowls exposed to −5 °C, the chickens fluff their feathers, crouch, and cover their legs to prevent heat loss [47]. Another manifestation is seen in birds such as hens that cover body regions such as the head, neck, and forelimbs with their feathers. Under hot conditions, those areas are essential routes of heat loss [57].

Another type of thermoregulation mechanism, non-shivering, is somewhat controversial. Recent evidence points to a relation between the up-regulation of the avian uncoupling protein (avUCP) and the capacity for non-shivering thermogenesis. Teulier et al. [59] evaluated this in one-week-old ducks (*Cairina moschata*) exposed to three environmental temperatures (25, 11, and 4 °C). They examined the expression of the avUCP gene together with the mitochondrial bioenergetics of the gastrocnemius muscle, reporting that the capacity for non-shivering thermogenesis depended on the environmental temperature, such that birds acclimated to low temperatures show greater efficiency in metabolic heat production parallel to positive avUCP regulation. This evidence establishes a direct relationship with the black-capped chickadees (*Poecile atricapillus*) studied by Milbergue et al. [37], who observed that food consumption increased by 44% under exposure to cold temperatures (−10 °C). Those authors attributed this to the possible positive regulation of cellular functions. Findings of this kind establish that in birds with greater muscle mass, such as domestic hens or turkeys, muscle movement is the adequate mechanism of thermogenesis, while in smaller species, alternate metabolic pathways of thermogenesis are found that entail energy costs [56,60,61].

Therefore, the presence of feathers in birds is controversial since their characteristics as heat insulators give them the ability to keep the birds in a thermal comfort zone. In contrast, their presence hinders heat loss in hot climates and makes them susceptible to heat stress. As Tattersall et al. [62] mentioned, the morphological characteristics that contribute to heat loss include the beak, a structure notable for its vascularization that allows heat exchange with the environment to vary due to great sensitivity to temperature changes concerning vascular contributions [29]. Comparing the temperature of the beak area with that of a limb in extreme climates revealed an inversely proportional relationship between these two measures. Other results indicate that extreme climate change can qualitatively influence thermoregulation by impeding predictions of responses in species [63]. These findings suggest that the limbs form the primary pathway for dissipating heat to the extremities and that the beak is a secondary pathway. However, the presence of other heat loss zones, such as the cloaca, cannot be ruled out. The absence of sweat glands in the bird’s body is probably related to the fact that the feathers would hinder evaporation from the skin and that moisture produced would wet the feathers reducing the heat loss efficiency and interfering with their flight function.

In related research, Hoffman et al. [64] evaluated the responses of Inca doves (*Columbina inca lesson*) and Eurasian quails (*Coturnix linnaeus*) when exposed to temperature increases. Cloacal evaporation was lower at temperatures of 30, 35, and 40 °C, but during exposure to 42 °C, an increase in cloacal evaporation compared to that of the beak and skin was observed. The authors reported the following distribution of evaporation: 58.2% dermal, 35.4% buccopharyngeal, and 6.4% cloacal, but that the latter showed a 14% increase under the extreme temperature, suggesting that the cloaca could be an emergency thermoregulation pathway. This observation has been questioned in comparative studies where caloric losses in birds exposed to heat stress are more significant in the oropharyngeal region than in the cloaca. However, the rate of the cloacal evaporation increases proportionally with the environmental temperature increase up to 40 °C, as a pathway for heat loss [62,63].

Additionally, although the cloacal region is suggested as a region for caloric loss in birds, many veterinarians currently avoid measuring cloacal body temperatures to prevent unnecessary stress on avian patients. Using IR thermometers in the axillary region provides a less invasive and reasonable measurement of core body temperature in birds to provide a more comprehensive health status assessment. For this reason, some studies have utilized IRT in the axillar region because that window has shown similar temperatures to the cloaca (40.35 vs. 40.38 °C) [65]. According to these results, options for determining the areas more sensibility to heat loss in animals with feathers include those where vasculature is proximal to the skin where it can facilitate heat conversion between blood vessels and the environment, as well as regions that are free of feathers, compared to other regions where the presence of feather may interfere with infrared readings.

In species with feathers, therefore, this body’s morphology and adaptive functions permit the strategy of insulating heat between the skin and feathers. While this trait is favorable in cold climates, it means that birds are susceptible to the effects of heat stress. Finally, the capacity of feathered animals to produce heat depends on the amount of muscle mass, as was determined in species that increase their cellular metabolism. During exposure to intense heat, the dissipation pathways in birds include the skin, beak, wattles, combs, and cloaca under extreme conditions.

### 2.2. Animals with Hair

**Figure 4:**
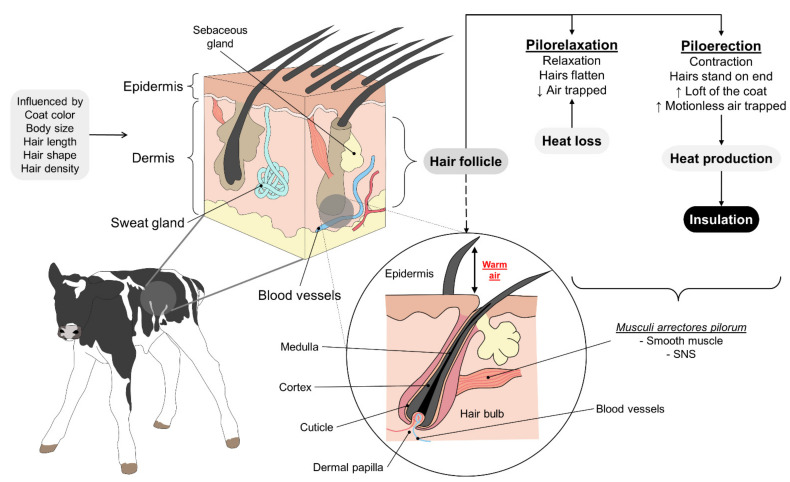
Hair coat and its effect on thermoregulation in mammals. The presence of hair and its characteristics (color, length, shape, density) play an active role in thermoregulation under extreme environmental conditions. Whether animals are exposed to cold or hot temperatures, the *musculi arrectores pilorum* controls the hair’s position to increase or decrease its insulation capacity through the action of the SNS. Pilorelaxation occurs in hot environments to flatten the hairs and reduce the amount of air trapped between skin and hair. This enhances heat loss and reduces body temperature. In contrast, under exposure to cold, piloerection increases the loft of the coat and, as a result, the amount of motionless air trapped. This action in mammals both produces and insulates heat to prevent any reduction of the core temperature. SNS: sympathetic nervous system.

The presence of hair gives animals a significant advantage in thermoregulation by preventing heat loss under cold conditions. Hair follicles are critical for thermoregulation because their vasculature comes from the deep dermal vascular plexus (Figure 4) [66]. The disposition of primary hair and secondary fibers are factors that affect the thermal insulation provided by hair or fur [67].

One example of this can be seen in the river buffalo. The skin of this species is rich in melanin, with high levels of absorbance, but because it has few hair follicles and sweat glands, these animals are susceptible to thermal stress due to the enormous amounts of solar radiation their bodies absorb under natural conditions [22,68]. This explains the adaptive behavior of buffaloes that prefer to stay in shady areas or wallow in muddy ponds as those conditions foster heat loss from the dermal surface [69,70]. Shade reduces the heat load and the direct solar radiation that the skin surface receives. On the other hand, wallowing reduces the animals’ body temperature that, combined with peripheral vasodilatation, dissipates heat into the environment through a combination effect of convection and evaporation. This process is somewhat similar to what happens in sweating [71]. In addition, the muddy layer that remains on the buffaloes’ back forms a protective layer against the sun’s rays and extends over time the cooling effect by interacting with the wind.

Meanwhile, fur’s degree of caloric insulation is proportional to its depth and thermal conductance [72]. Differences in the conductivity of fur vary with species and inter-layer density. For example, layers with 100–200 fibers/cm^2^ are thick and straight, so their heat insulation capacity is moderate [73]. Jørgensen et al. [74] worked with horses and examined the differences in the hair of 21 cold-blooded and warm-blooded animals. The latter had shorter hair and presented higher surface temperatures measured by IRT (*p <* 0.05). Those authors also included donkeys, mules, and horses, comparing their hair’s weight, length, and width. Despite the similar evolutionary history of these species the characteristics analyzed presented particularities. The donkeys did not show differences in these hair measurements between the year’s seasons, but their hair layer was lighter, shorter, and thinner than horses, suggesting that donkeys have low adaptability to cold climates [75]. These results demonstrate the importance of considering the breed of the animals studied and their age since hair density undergoes changes over time that explain the difference in resistance to wind penetration between adult and young animals since older ones tend to have thicker coats [76]. These studies also underscore the importance of identifying the differences between animals that occur in cold versus warm climates.

In Arctic species, the presence of hair and reserves of adipose tissue, mainly subcutaneous fat, play fundamental roles in maintaining homeothermy. Soppela et al. [77] evaluated the thermoregulation capacity of young Finnish reindeer (*Rangifer tarandus* L.) aged 1–35 days. Larger animals tend to have thicker fur than smaller ones. Relative body surface area has an allometric relation with body mass with an exponent of −0.33, and a specific heat conductance has an allometric relation with body mass and an exponent of −0.50. Relative body surface area has an allometric relation with body mass with an exponent of −0.33. Specific heat conductance has an allometric relation with body mass and an exponent of −0.50. The mass exponents in these two expressions show that conductance decreases more rapidly with increasing body size than does body surface area, so insulation is greater in the larger animals [78]. The winter fur coat of this species consists of thick layers of guard hair with cavities that fill with air and an additional layer of woolen hair. Though housed at an environmental temperature of −22.5 °C, those animals maintained euthermia as measured by rectal temperatures of 39–41 °C, thanks to the process of thermogenesis effectuated by sympathetic noradrenergic activity on brown adipose tissue in response to cold. In contrast, when exposed to temperatures of +20 °C, the animals increased their rectal temperatures by as much as 1 °C. That study also compared the density and length of the thickest layer of hair between adults and calves. The adult animals showed variations among different body zones: 2000/cm^2^ and 12 mm in the legs, 1000/cm^2^ and 30 mm in the abdomen, and 1700/cm^2^ and 30 mm in the back, respectively. In contrast, the dorsal region of the calves had a density of 3200/cm^2^ with a length of 10 mm.

**Figure 5:**
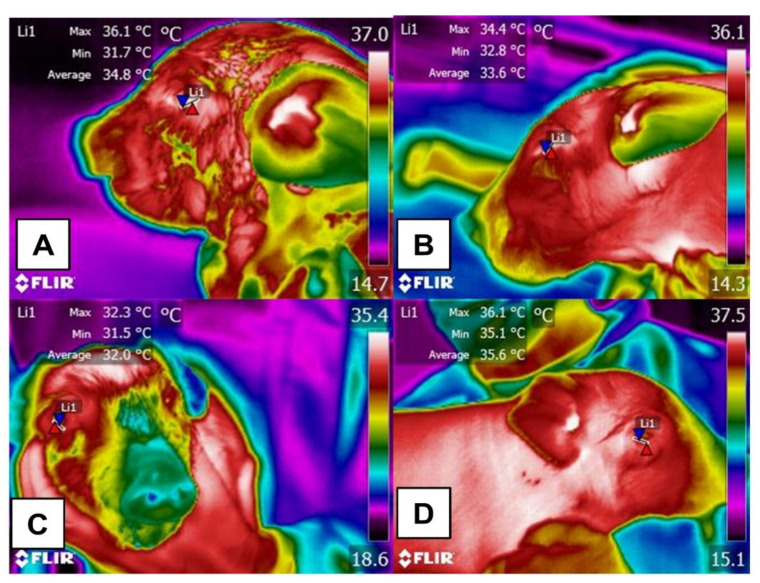
Effects of skin with fine hair on lower eyelid dermal microcirculation in a newborn piglet under four conditions: moist, dry, and before and after ingesting colostrum. (**A**) moist newborn piglet. While moist, this piglet has an average upper eyelid temperature (Li1) of 34.8 °C (with maximum and minimum temperatures of 36.1 and 31.7 °C), possibly due to heat preserved from the covering of amniotic fluid that allows it to maintain a stable temperature. (**B**) dry newborn piglet. The absence of hair, low thermoregulating capacity, contact with cold surfaces, and moisture cooling from the amniotic fluid contributed to heat loss through evaporation. A decrease of approximately 1.7 °C, with maximum and minimum temperatures of 34.4 °C and 32.8 °C, was observed in this animal. (**C**) dry piglet before consuming colostrum. Although the piglet is dry, an average decrease in the maximum and minimum temperatures of 2.1 °C is seen. In this case, the absence of hair, the limited thermogenesis capacity of newborns, and direct contact of the skin with cold objects contributed to heat loss through convection. (**D**) dry piglet after colostrum ingestion. An increase of approximately 3.8 °C was recorded, a temperature similar to the maximum one recorded in thermogram (**A**). This suggests a thermogenic response attributable to colostrum ingestion that aids in homeostasis and thermoregulation in piglets.

These observations are similar to those seen in young harp seals (*Pagophilus groenlandicus*), where the presence of a layer of short, flat hair plays an essential role during thermoregulation. In these animals, the fur has nerve fibers that, when activated, utilize adipose tissue when exposed to cold environments [79]. The skin of young seals has low thermal resistance, but this increases with the transition from fur to fat [80]. It is important to mention that conduction capacities under different thermal conditions can vary depending on contact with water or air. Fur tends to flatten as it comes into contact with water, providing greater thermal conductance in the skin underwater than when exposed to air [79]. Figure 5 illustrates the influence of cold water and air according to studies carried out by the authors about the thermal conductivity of hair and fur.

In animals exposed to intense heat or warm climates, dense fur protects them from absorbing high solar radiation levels through the dermal surface [81]. In hot environments with intense solar radiation, the coat seems to play a paradoxical role. The coat must have high conductance allowing the speed of centrifugal heat flow and promoting heat loss. On the other hand, the same coat should be an obstacle to the entry of centripetal heat fluxes, making heat acquisition by the animal more difficult. In some cases, piloerection can help aid in heat loss by raising the hair through the action of the musculi arrectores pilorum. This produces insulation for the body and generates immobile air near the skin surface [82]. Hair, therefore, functions as an insulating surface that protects animals from the intense heat characteristic of this type of environment. This action is due to the piloerection effect that promotes caloric loss. Although a similar event also occurs in feathered animals to intensify heat loss, it is more effective for radiated heat than dry heat insulation [83]. However, this aspect is not linear. In herbivores, increasing the thickness of the coat, increasing the insulation, whether or not due to piloerection, does not per se reduce the heat acquired or heat loss. Often the opposite is true. Thicker coats are often responsible for more energy received by the epidermis. These coats usually have higher transmittances, and shortwave radiation is transmitted along with thick coats in larger quantities. To this condition, it is essential to mention that thicker coats are related to the lower efficiency of evaporative heat loss. Often the sweat evaporates but remains inside the coat, increasing the partial vapor pressure and thermal conductivity.

In this field of research, Wacker et al.’s [84] study of dunnarts—*Sminthopsis crassicaudata*, a species with dark bands that serve as camouflage—analyzed the relation between the function of those bands and the animals’ thermal characteristics. The authors found a positive correlation between exposure to solar radiation and skin temperature underneath the pelt (*r*^2^ = 0.59), an inversely proportional relation between resting metabolism and the length of the dark bands (*r*^2^ = 0.77), and a lower energy requirement for reheating (*r*^2^ = 0.75). These colorimetric characteristics of the fur provide an efficient camouflage for the environment and a means of conserving radiation.

As these cases show, the function of hair changes by environmental conditions, as a thermal sensation of cold tends to insulate the heat produced by the metabolism of adipose tissue, while in warm climates, fur can help dissipate heat through strategies such as piloerection. Furthermore, thermal conductance can be affected by the presence of water or wind and the inherent characteristics of animals, including breed, age, and fur color.

### 2.3. Animals with Glabrous Skin or Fine Hair

As discussed above, as a way to protect themselves from potential damage caused by intense thermal heat or cold, or direct solar radiation, animals have developed body coverings such as feathers or hair that help control heat exchange between their bodies and the environment [72]. However, dermal microcirculation governed by the most superficial dermal capillaries in animals with glabrous skin is a key thermoregulation strategy for conserving or dissipating heat [12,85]. The effects of this feature acquire importance for these species during the first hours, days, or weeks of life because, during those periods, the thermoregulating capacity of these animals is deficient to such a degree that it can sometimes result in the death of newborns [35]. Given their inability to efficiently control internal mechanisms to maintain optimal body temperature in cold environments, these animals adapt postural changes to prevent heat loss, such as reducing contact with cold surfaces, snuggling, and producing heat through muscular contractions, as Figure 2 shows. This helps us understand the importance of hair. In some breeds of dog, anhidrotic ectodermal dysplasia, a type of congenital, multifocal alopecia that affects hair development, reduces their thermoregulating capacity because it is characterized by the complete absence of hair follicles, the piloerector muscles (arrector pili muscle), and sebaceous and apocrine glands [86]. This dermal disease deprives dogs of protective hair, leaving the skin exposed to all manner of thermal environmental injury, including exposure to air currents and/or contact with cold surfaces, as often occurs in neonates [87]. However, these behavioral strategies for thermoregulation are not these animals’ only means of preventing the heat loss caused by their lack of fur, for producing heat by consuming energy resources is also valuable. Studies of piglets have reported that birth weight and its increase following birth have a positive relation (*r*^2^ = 0.75) related to colostrum ingestion [88], an alimentary substance that provides newborns with thermal stability. A later study confirmed this, demonstrating that consuming colostrum stabilizes temperature by raising the piglets’ metabolic rate. However, if the neonates’ thermal environment is inadequate, their colostrum ingestion will decrease, limiting the advantages that this food provides (Figure 5) [89]. Research on pigs also shows that the Suidae family has a functional loss of the brown adipose tissue (BAT). The BAT is considered a thermogenic organ by activating specialized proteins (uncoupling protein 1) in other species such as rats, sheep, and even humans [90]. BAT activation is mainly due to a hypothalamic-sympathetic response that activates the adipocytes through norepinephrine [91]. However, in pigs, the lack of functional BAT makes them susceptible to cold stress, and it is suggested that the primary mechanism of thermogenesis depends solely on shivering from the skeletal muscle [92]. This effect is of particular interest in newborn piglets, who, despite depositing large amounts of fat in subcutaneous tissue (15%), require shivering to produce heat due to the BAT lacking and the low amount of white adipocyte tissue [90].

In pigs, another factor related to their morphology is the skin structure. Skin thickness depends on breed, so some are better adapted to life in warmer climates than others. In their work with large white European and Caribbean creole pigs, Renaudeau et al. [93] reported that the thickness of the dermis is also a trait that provides greater resistance to cold conditions. Those authors found that the skin thickness in the dorsal region of 20 Caribbean creole animals was significantly greater than in 20 large white Europeans (3.60 vs. 3.13 mm; *p <* 0.01, respectively), accompanied by a higher density of sweat glands (32.0 vs. 25.4 glands per mm^2^; *p <* 0.01), though the surface of those glands was smaller. These characteristics were associated with adequate dissipating heat in the Creole breed and its high heat tolerance in hot climates.

Another hairless animal species, the naked mole-rat (*Heterocephalus glaber*), has significant differences in the anatomical characteristics of the dermis compared to the common mole-rat (*Cryptomys hottentotus*). Although the dermis of these two animals is structurally similar, the deepest internal layers where adipose tissue (panniculus adiposus) is deposited form an insulation site that is present in all hairless mammals. However, this is not a normal and efficient mechanism of thermal regulation. In addition, the epidermis of these rodents is thin, contains larger blood vessels, and has a loosely folded morphology, all of which exacerbate its inability to regulate body temperature [94].

Birds, of course, are characterized by having feathers, but some species have areas of glabrous skin that have been interpreted as a thermoregulation mechanism. The presence of patches of glabrous skin, such as the bald head of vultures (European griffon, *Gyps fulvus*), is a trait associated with an adaptive improvement in response to the extreme temperatures to which those birds are exposed (40–70 °C at ground level with decreases of 6.5 °C for every 1000 m of flight height). However, in the case of birds, postural changes are also important. To dissipate heat, birds may fully extend their neck and feet and spread their wings while adopting a seated posture and covering their bare feet, breast, neck, and part of the head with their feathers are means of conserving heat. A mathematical model developed by Ward et al. [95] suggests that these behavioral responses can affect the percentage of glabrous skin exposed to the environment in a range of 7–32%.

Exposure to water at different temperatures is another factor that can induce physiological and biochemical changes. In this case, concentrations of circulating catecholamines and immersion times will modify the organism’s responses and behavior. One study in this field determined that an exposure time of 30 min generates an increase in heart rate (HR), while blood pressure tends to decrease to keep the body temperature at 38.4 °C [96].

In contrast, when an increase in heat production is required, or under the effects of a warm climate, the goal of the peripheral physiological response is to dissipate heat [97]. Here, central vasoconstriction begins to decrease progressively at 41 °C [11]. This circulatory effect is important clinically because internal temperatures above 42 °C can cause cerebral ischemia, as studies of rabbits exposed to those conditions have shown [98]. These dermal vascular responses involve the sympathetic nervous system via signaling by cardiopulmonary baroreceptors [99]. Mack et al. [100] determined that neurochemicals such as the calcitonin gen-related peptide (CGRP) act during cutaneous vasodilatation to produce local warming and raise temperatures to 38 °C. That study also reported that at a dermal surface temperature of 42 °C, there were no modifications of CGRP, despite a proportional increase in thermal stress (*r*^2^ = 0.62). Therefore, vascular responses are more important in regulating temperature in animals with glabrous skin because thermal exchange occurs more quickly than in animals with protective layers.

## 3. Use of IRT as a Minimal Invasive Technique for Detecting Temperature Differences in Animals under Conditions of Heat or Cold Stress

Current technological development in this field is focused on reducing the environmental factors that challenge the capacity in human-controlled operations. This requires continuous temperature monitoring to predict handling stress and inflammatory pathologies and to implement therapies opportunely [101]. In this context, one of the most efficient, non-invasive methods for evaluating temperature signals is IRT [102] because this tool allows us to assess thermal changes on the skin surface due to modifications in the microcirculation [103]. In the beginning, the use of this technology was limited to detecting the heat produced by inflammatory pathologies [104], the overheating of surgical equipment to prevent thermal damage to adjacent tissues during operations or evaluating changes in surface temperature during painful events [103]. Currently, it has been suggested that the evaluation of the surface temperature in some anatomical regions can provide information about the core temperature, as has been reported by Fiebig et al. [1] who evaluated the rectal temperatures of 10 naked male rats over 14 days using three non-invasive methods (subcutaneous, intraperitoneal, rectal) and IRT did not detect any significant differences among the measurements recorded. Therefore, IRT could be used to determine, non-invasively, physiological parameters such as the core temperature.

Results to date of the use of IRT with humans are controversial. In one study of 200 children aged 1–4 months, IRT underestimated the temperatures of patients with fever and overestimated the number of patients without fever (*r* = 0.48) [105]. On the other hand, infrared readings of the lateral side of the neck showed a sensitivity of 95.5% to 78.8%, in contrast to the forehead, where 11.4% of 184 febrile children were not detected by this non-invasive method [106]. These findings demonstrate that IRT could be a reliable tool to assess febrile states in children, but the accuracy of the IRT depends on the selected thermal window [107].

In veterinary medicine, IRT permits evaluating hypothermia in thermo-sensible species such as newborn piglets, where the low energy reserves and absent BAT reduces their thermogenesis activity [108]. Other studies have focused attention on identifying the most suitable method for measuring heat loss in lambs. One evaluation of shorn and unshorn newborn lambs subjected to an experimental cold test for 1–4 h post-birth showed that surface temperatures decreased gradually over time, though it was not possible to identify why the temperature in the hip region was 0.7 °C higher than in the shoulders of the shorn lambs [109]. Furthermore, in another study with 85 neonatal lambs, although the IRT detected heat loss (between 32.7 and 24.4 °C) due to environmental factors (cold or wind) and wet body surface, this tool could not be related to rectal temperature within the first 24 h [110]. This effect is attributed to thermogenesis the first 1 to 5 h of birth and depends on the activation of brown fat. However, when this period is exceeded, thermoregulation depends mainly on the colostrum intake [110].

In adult sheep, in whom the presence of fleece implies a mechanism to adapt to extreme environmental conditions, Pantoja et al. [111] evaluated the effect of climate change and heatwaves and their influence on the superficial temperature of animals located in areas with tropical climates. When evaluating the body surface of rams of the Morada Nova, Santa Inés, Dorper, and Texel breeds for one year, the authors found that the temperature increased depending on the time of the day (higher at 13:00 to 20:00), and during warm weather seasons (maximum environmental temperature of 31.2 °C) in the eyeball region, testicular region and left antimera region, but different values were registered. While the orbital temperature did not change significantly (*p* > 0.05), the temperature at the thoracic and abdominal regions showed differences between genotypes. Their surface temperatures were low in the Texel genotype, whose wool is longer, has a thick coat, and white color. In contrast, in Santa Inés sheep, who have short hair and black fur, the IRT values increased up to 5.5 °C in the back region during the summer. A similar result regarding heat tolerance and genetic differences between races has been reported by Pulido-Rodríguez et al. [112]. In 30 sheep of the Santa Inés, Dorper × Santa Inés (hair), and White Dorper × Santa (wool) Inés breed, it was found that in animals with white wool, the dorsal, ventral, and shoulder IRT was higher than other genotypes. Wool limits heat loss by evaporation, causing altered respiratory patterns due to the insufficient capacity to dissipate the heat.

This thermoregulatory effect and the use of IRT in farm animals has also been evaluated in dairy goats, where morphological characteristics such as skin pigmentation influence the ability of the animals to maintain their thermoneutrality. Darcan et al. [113] determined in 26 cross-bred Saanen goats that pigmented animals had higher surface temperatures of the head and udder (above 37 and 37.5 °C), compared to poorly pigmented goats (range between 32.45 ± 0.21 to 37.49 ± 0.21 °C). Ten five-year-old goats were subjected to acute heat stress in tropical environments to assess IRT in the same breed’s dorsal, tail, eye, and mammary gland IRT. It was found that IRT had a significant correlation with rectal temperature (*r* = 0.94, *r* = 0.87, *r* = 0.92, and *r* = 0.93, respectively), managing to differentiate between animals that had been in low, severe, and high thermal stress mainly due to direct solar radiation [104]. This was reported in Anglo Nubian, and Alpine brown goats reared in semi-arid regions concerning breed differences in goats. Through IRT, no significant interaction was reported due to genotype (*p* < 0.05); However, when evaluating physiological parameters such as respiratory rate, the increase in the rate recorded in alpine goats suggests that Anglo-Nubian animals are more adapted to the mentioned climatic conditions [114].

IRT stands out for its usefulness in identifying the region’s increased surface temperature due to diverse causes, including pathologies and physiological states. However, we must recognize that its validity depends on climatic conditions, the region to be evaluated, the species, and the presence of the integumentary system (with or without glabrous skin, hair, or feathers), all of which can alter the interpretation of results. In the same way, technical elements such as the shooting distance, the type of lens, and the reflectance need to be considered when using IRT.

## 4. Effect of Hair, Feathers, and Glabrous Skin Based on Recent Scientific Findings on the Use of IRT

### 4.1. Hair

**Figure 6:**
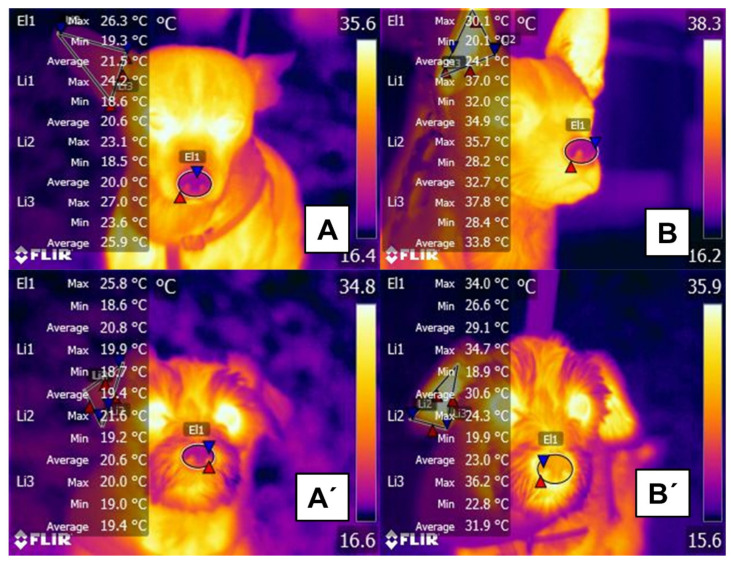
Effect of exercise (running for 15 min) on changes in dermal microcirculation in short- and long-haired dogs. (**A**) rostral region of a short-haired dog at rest. The maximum nasal temperature (El1) is 26.3 °C, and the maximum right ear temperature (Li1, Li2, Li3) is 27 °C. (**B**) rostral region of a short-haired dog after running. Despite the similar color pattern to thermogram (A), an increase in the maximum nasal (El1) and auricular temperatures (Li1, Li2, Li3) was recorded (3.8 °C and up to 10.8 °C, respectively). (**A’**) rostral region of a long-haired dog at rest. The maximum muzzle temperature (El1) is 25.8 °C, 0.5 °C lower than in the short-haired dog. The maximum temperature of the right auricular (Li1, Li2, Li3) region is 21.6 °C. (**B’**) rostral region of a long-haired dog after running. Compared to thermogram (**A’**), an increase of 8.2 °C was registered in the maximum temperature of the nose (El1), seen in the color change in that region. In contrast, the temperature of the pinna (Li1, Li2, Li3) was 1.6 °C lower than in the short-haired dog (maximum value 36.2 °C). These findings suggest that the cutaneous vasodilatation response in the nasal region is greater in long-haired than short-haired dogs, while the latter present more evident vasodilation responses in the pinna. The higher surface temperature observed in dogs with short fur is mainly related to the closeness of the epidermis to the fur surface, which makes it possible to evidence more increased energy flow by longwave radiation. In the case of the dogs being under direct solar radiation, the situation could be inverse, depending on the thickness and color of the fur. Being darker will determine more significant absorbance and minor transmittance [115].

The presence and influence of hair on heat dissipation has emerged as the principal focus of many studies. A recent attempt to determine the effect of fur type on surface temperature evaluated 50 dogs with different coat types (curly, short, long, and double) by examining the entire left and right lateral regions. The animals with short fur presented a higher temperature (31.3 °C) than the dogs with long fur (28.3 °C) and the double layer (28.2 °C), while those with curly hair had an intermediate temperature (29.8 °C). This suggests that animals with a layer of short hair tend to present a higher surface temperature increase than those with a layer of long hair [115]. These differences are presented in Figure 6.

Another way of interpreting this effect could be due to a reduced capacity of the short-haired dogs to prevent heat gain and loss [72]. These observations coincide with those of Autio et al. [116], who evaluated nine weaned horses in a free-roaming environment in wintertime, finding a heat loss greater than −16 °C. The trunk region presented a temperature of −23 °C due to the presence of frost. Although the authors sustain that IRT is not an adequate tool for evaluating heat loss, their opinion reaffirms the need to identify a reliable thermal window or region of interest that clearly indicates the thermal state of individuals.

Reyes-Sotelo et al.’s [87] recent review of IRT suggests implementing this tool to monitor the surface temperature of neonate dogs, where it is vital to prevent heat loss in the early stages. Somewhat similar to human phenomena, when used during neonatal care thermal images can identify general thermal physiology and heat loss [117]. Observations of human newborns show that the temperature of the thoracic region has values similar to those of body temperature and that this correlates significantly with parameters such as the mother’s weight (*r*^2^ = 0.31) [118]. This has also been demonstrated in neonate dogs, where alterations in blood flow permit evaluating the risk of death during normal births [119].

Likewise, studies in outdoor conditions must consider the impact of the heat generated during physical exercise. This effect was observed in Jack Russell and miniature Pinscher dogs after 15 min of walking, 10 min of trotting, and a period of running. That study showed that readings from the shoulder, flank, and back increased significantly compared to the rectal temperature. This suggests the need to consider fur color and its influence on heat production when utilizing IRT. In the absence of solar radiation, surface temperature results primarily from longwave radiation emitted by the animal. However, in outdoor conditions, the surface temperature is related also to incident short wave radiation and the fur reflectance, absorbance, and transmittance, depending on the fur’s color and thickness [34].

### 4.2. Feathers

**Figure 7:**
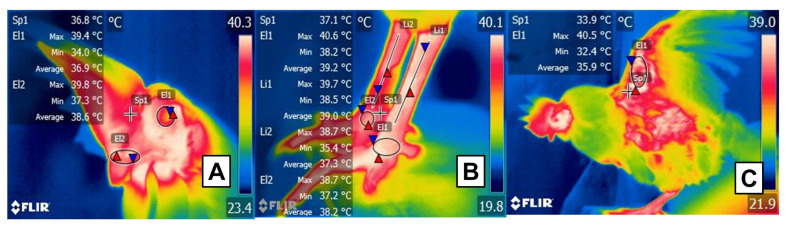
Potential thermal windows in birds. (**A**) Facial region: marked by a circle (El1) and limited to the orbital region, including the upper and lower eyelids. The cere and nares of birds (El2) are suggested as a potential window that participates in heat dissipation due to vascularization through the maxillary artery. Sp1 shows the default focal point of the software. (**B**) Plantar region: the tibial (Li1, Li2) and plantar region (El1, El2) receive vascular supply from the femoral and dorsal metatarsal arteries that contribute to thermal exchange. Sp1 shows the default focal point of the software. (**C**) Radial region: due to the presence of the radial artery that irrigates the axillary region (Sp1), this window incorporates the inner face of the wing (El1) into evaluations.

Studies of animals with feathers using invasive techniques have correlated circulating cortisol levels with the ocular region’s temperatures under caloric stress conditions (Figure 7) [31]. It should be noted that the ocular region is widely used in domestic animals to determine the increase in body temperature in response to the activation of the sympathetic nervous system under stressful conditions such as stress hyperthermia [120]. This effect is observed as an increase in the superficial temperature of the orbital region [121,122].

However, increases in the IRT of the orbital region have not only been associated with stressful stimuli. In studies in laying hens, it was observed that the animals exposed to environmental enrichment, to improve their well-being, the temperature of the ocular surface and the levels of corticosterone were higher, in comparison with animals that did not receive any enrichment [123]. Regarding this, Jerem et al. [124] mention that IRT, together with other techniques such as hormonal determinations, is an alternative to identify and correct potentially stressful situations for this species.

On the other hand, Powers et al. [125] used IRT to assess passive heat dissipation in facial regions of various species of hummingbird (*Cynanthus latirostris*, *Archilochus alexandri*, *Eugenes fulgens*) during flight, as well as in smaller species (*Lampornis clemenciae*, *Selasphorus calliope*). They observed that the latter had higher temperatures and lacked the mechanism of passive heat dissipation even when inactive. They also mention that this characteristic could justify using thermal shelters to benefit wild and domestic birds during rest to prevent heat loss [126].

Loyau et al. [127], in contrast, sustained that resistance to heat stress in domestic species can be improved through the genetic selection of laying hens. They evaluated heat dissipation through the wings based on 9355 IRT images which showed that the temperature of the shank presented higher estimates of heritability (0.22) than the wing (0.09) or comb (0.19). That correlation was attributed to the genetic selection of individuals. Additional essential data from that study revealed that the wing is a thermal window reflecting environmental temperature, so evaluations of featherless areas are necessary to determine the usefulness of IRT in this species and identify adequate thermal windows for its use.

### 4.3. Glabrous Skin

In the case of species with glabrous skin, interest centers on heat dissipation that occurs during exposure to cold. The principal concern with domestic pigs, for example, is the farrowing period when the presence of moisture can negatively impact neonates during the first hours of life [128,129]. Wet surfaces have higher thermal conductivity coefficients, which leads to a substantial increase in the rate of heat loss of neonates, which have a high specific surface area and high conductance. Moreover, evaporation of moisture from the dermal surface and the resulting increase in heat loss can trigger hypothermia, which is detectable by IRT [130]. This indicates a potential role for IRT in clinical and preventive veterinary medicine since it could be utilized in production units to reduce the mortality risks in this species.

For example, Schmitt et al. [131] used the IRT to evaluate the neonatal survival of genetically selected piglets (animals with low residual feed intake and high residual feed intake). In 62 piglets, thermography associated with standard methods such as rectal temperature was shown to be a reliable predictor of postnatal thermostability in the first hour after birth, a period with high mortality rates due to hypothermia. Likewise, IRT identified that the animals with low residual feed intake had better adaptability to heat stress and that only the back and the ear base temperature correlated with rectal measurements.

## 5. Perspectives on the Use of IRT and Species-Specific Thermal for Animals with Distinct Integumentary Structures

Using IRT, the evidence discussed above highlights the importance of evaluating temperature in animals at the central level and on the surface—a tool that can provide information on the physiological and behavioral responses that animals adopt in extreme climates and permit the detection of peripheral circulatory changes caused by infectious and inflammatory pathologies [69]. It is advisable to implement continuous, automated temperature monitoring of production animals or those in clinical conditions because it can opportunely quantify alterations in the temperature of animals and associate them with pathological processes and physiological parameters such as the heart or respiratory rate [132,133,134]. It is, however, necessary to perform additional studies of IRT’s validity and applicability in the real environments of diverse species.

As this document suggests, one possible limitation of applying IRT is the presence of hair, feathers, or glabrous skin because these structures alter thermal exchange and affect the levels of radiation that bodies emit in extreme climates. These elements need to be considered to interpret the thermal states of animals correctly [69]. In addition, the anatomical and morphological differences highlighted in this paper show the need to validate specific thermal windows for different species—and even different breeds of the same species—since research shows that the type of protective layer, its color, density, length, and form, can all influence interpretations of IRT images [135,136].

## 6. Conclusions

Temperature, a vital parameter of all animal species and breeds, is influenced by diverse external and internal factors that modify organisms’ physiological and behavioral responses as they seek to adapt to different environments. In some species, structures of the integumentary system, such as skin, hair, and feathers, strongly affect the thermoregulation capacity of individuals and, as a result, the amount of heat they conserve or dissipate through their bodies. IRT, a tool that quantifies the infrared radiation emitted by a body, depends directly on these anatomical-morphological differences to objectively measure body surface temperatures in diverse species.

Because birds’ feathers function as insulators, measuring irradiated temperature in feathered zones is impossible. However, birds also have featherless areas such as the feet, beak, orbital region, and the head or breast of species such as vultures that can be considered potential thermal windows for evaluation. In contrast, hair, a characteristic of most mammals, presents diverse challenges due to variations in density, length, color, and form. This means that animals of the same breed but with different kinds of hair will have thermoregulatory mechanisms that are more or less efficacious than others. This fact directly affects IRT measurements.

In contrast, in hairless animals, the entire body may be considered a thermal window because microvascular changes in these species can be detected easily by thermography. However, this condition also makes them more susceptible to extreme climates since they do not have a layer to protect them from those conditions. Furthermore, in sweat-intensive glabrous animals, when subjected to active convective processes, they may show sufficiently high vaporization rates that can reduce skin surface temperature. For this reason, IRT has been proposed as a valuable technique for identifying hypothermia before hemodynamic imbalances can occur, potentially with severe repercussions for animal health.

Concerning the scope and perspectives for the use of IRT, based on the corpus of evidence consulted, we conclude that it helps evaluate the surface thermal responses that occur in response to changes in environmental temperature and in cases of pathology, intense exercise, and other states where it can function for early diagnoses in domestic animals. Finally, a note of caution: it is essential to consider each species’ specific morphological characteristics and physiological mechanisms to be evaluated.
